# Severe Malaria and Artesunate Treatment, Norway

**DOI:** 10.3201/eid1411.080636

**Published:** 2008-11

**Authors:** Kristine Mørch, Øystein Strand, Oona Dunlop, Åse Berg, Nina Langeland, Rafael A.M. Leiva, Jørn-Åge Longva, Håkon Sjursen, Steinar Skrede, Jon Sundal, Mogens Jensenius

**Affiliations:** Haukeland University Hospital, Bergen, Norway (K. Mørch, N. Langeland, R.A.M. Leiva, J.-A. Longva, H. Sjursen, S. Skrede); Akershus University Hospital, Nordbyhagen, Norway (Ø. Strand); Ullevål University Hospital, Oslo, Norway (O. Dunlop, M. Jensenius); Stavanger University Hospital, Stavanger, Norway (Å. Berg, J. Sundal); 1 Current affiliation: Aalesund Hospital, Aalesund, Norway.

**Keywords:** Plasmodium falciparum malaria, artesunate, artemisinin, letter

**To the Editor:** Approximately 8,000 cases of imported falciparum malaria are reported in Europe each year (*1*). In a study from Belgium of 1,743 persons with fever acquired in the Tropics, only falciparum malaria resulted in deaths (*2*).

Until recently, the standard treatment of severe malaria was intravenous quinine (*3*). Frequent adverse effects, however, and reports of limited clinical efficacy in some falciparum malaraia–endemic areas preclude its usefulness (*4*). In contrast, artesunate, a water-soluble artemisinin derivate extracted from the plant *Artemesia annua* (quinghao), is considered safe and highly efficacious (*4*,*5*). Artesunate has the advantage of rapidly killing malaria parasites only a few hours after invading the erythrocyte, and it also reduces cytoadherance (*4*). Resistance to artesunate at the Cambodia–Thailand border has been reported, but until now artesunate resistance has not been considered a problem in most malaria–endemic regions (*5*,*6*). On the basis of 6 randomized controlled trials comparing artesunate and quinine, a recent Cochrane review recommended artesunate as the first-line treatment in adults with severe malaria in such areas (*7*). Similar recommendations were issued by the World Health Organization (WHO) in 2006 (*8*). Also, the European surveillance network, TropNetEurope, and the Advisory Committee on Malaria Prevention in UK Travelers advocate artesunate as the first-line treatment for severe falciparum malaria in travelers (*9*,*10*). However, the manufacturers of intravenous (IV) artesunate have not achieved Good Manufacturing Practice certification; currently, the drug is not widely used outside Asia.

In March 2008, an inquiry for patients treated with IV artesunate for severe falciparum malaria was mailed to all major departments of infectious diseases in Norway. All departments responded, and 9 patients treated from February 2006 to May 2008 were identified at 3 centers: 7 at Haukeland University Hospital in Bergen, 1 at Akershus University Hospital in Nordbyhagen, and 1 at Ullevål University Hospital in Oslo. Clinical and laboratory features were retrospectively obtained from the medical records. Artesunate was produced by Guilin Pharmaceutical, Guangxi, China, and provided from IDIS Pharmaceutical, Weybridge, United Kingdom.

With the exception of 1 patient who had become infected while in Myanmar, all patients acquired falciparum malaria in West Africa (Table). Four patients were Norwegian tourists or businessmen; 4 patients were visiting friends and relatives and had lived in Norway for 2, 15, 20, and 40 years, respectively. One patient was a pregnant (third trimester) immigrant. None of the patients had used antimalarial chemoprophylaxis. The patients’ symptoms fulfilled up to 5 of the WHO criteria for severe malaria: 1 patient had cerebral malaria, 5 impaired consciousness, 5 jaundice, 2 shock, 2 renal failure, 2 hemoglobinuria, 1 hematemesis, and 8 hyperparasitemia (Table). The initial treatment consisted of IV artesunate plus doxycycline (n = 7), IV artesunate monotherapy (n = 1), or IV artesunate plus clindamycin (n = 1). The dosing of artesunate was 2.4 mg/kg at 0 h, 12 h, and 24 h and then daily thereafter. Patient 6 received a 1,200-mg loading dose of quinine before transfer to one of the study hospitals (Table). None of the patients needed exchange transfusions. No adverse effects were attributed to artesunate, and the pregnant patient delivered a healthy child at term. The parasitemia level fell below 1% in all patients within 1–2 days. Treatment was changed to oral antimalarial drugs (artemether–lumefantrine, mefloquine, proguanil–atovaquone, or quinine) within 2.1 days (mean); all patients recovered uneventfully and were discharged from the hospital within 4.2 days (mean) (Table). No episodes of recrudescence were documented posttreatment at 4 weeks follow-up; 7 patients had a negative malaria slide and 2 patients were not examined for parasites but had no clinical recrudescense at follow-up.

**Table:** Epidemiologic, clinical, and laboratory data from 9 patients with severe falciparum malaria treated with intravenous artesunate, Norway, 2006–2008*

Patient no. (gender/ age, y)	Reason for travel	Country of disease acquisition	WHO severe malaria criteria	Days from symptom onset to therapy	Initial treatment						Length of hospital stay, d
						Parasitemia level, %					
						Day 0	Day 1	Day 2	Day 3	Day 4	
1 (M/37)	Tourism	Ghana	Impaired consciousness, bilirubin† 53, hyperparasitemia	10	AS + D	4	<1	0‡			4
2 (M/45)	VFR	Mali	Hyperparasitemia	4	AS + D	5	<1‡	<1	NA	0	4
3 (M/25)	VFR	Ghana	Impaired consciousness, hematemesis, hemoglobinuria, lactate 3.2,§ hyperparasitemia	5	AS + D	15	7	<1	0‡		3
4 (M/41)	Tourism	Ghana	Coma, shock, hemoglobinuria, bilirubin 241, hyperparasitemia	5	AS + D	20	3	<1	NA	0‡	5
5 (F/32)	Immigration	Nigeria	Impaired consciousness, bilirubin 50, hyperparasitemia	3	AS + C (patient pregnant)	7	0	NA‡			3
6 (M/46)	Business	Nigeria	Impaired consciousness, creatinine† 309, bilirubin 58, hyperparasitemia	6	Quinine 1,200 mg loading dose, then AS + D	30	5	0.5‡	0		7
7 (M/35)	Tourism	Myanmar	Impaired consciousness, hyperparasitemia	10	AS	4	<1	0‡	0		5
8 (F/38)	VFR	Liberia	Shock	7	AS + D	1	<1‡	0	0		3
9 (M/55)	VFR	Guinea	Creatinine 315, bilirubin 118, hyperparasitemia	4	AS + D	6	<1	0‡			4

*WHO, World Health Organization; AS, artesunate; D, doxycycline; VFR, visiting friends and relatives; NA, not available; C, clindamycin. †µmol/L (bilirubin reference range 5–25; creatinine reference range 60–105). ‡Day when intravenous artesunate was discontinued. §mmol/L (reference range 0.5–2.2).

Our findings support those of several randomized controlled trials performed in Asia and indicate that therapy with IV artesunate is safe, induces rapid parasite clearing, and usually results in swift clinical cure. Blood exchange transfusion, a labor-intensive and potentially hazardous procedure, was initially considered for 2 of our patients but was deemed unnecessary because of the rapid improvement after artesunate treatment. Artemisinins have short half-lives, and there is an increased risk for recrudescence if used alone. We gave concurrent IV doxycycline or clindamycin to all but 1 of our patients; all patients were treated with an oral drug after IV artesunate, and recrudescence was not noted.

A major obstacle for the use of IV artesunate is its poor availability outside Asia and the fact that its use is not approved in many countries. However, in the United States, artesunate for infusion may now be obtained as an investigational drug from the Centers for Disease Control and Prevention (www.cdc.gov/malaria/features/artesunate_now_available.htm), and in the European Union, artesunate recently received the Orphan Medicinal Drug Designation from the European Medicines Agency (www.emea.europa.eu/pdfs/human/comp/opinion/48693207en.pdf) and may be obtained from IDIS Pharma (www.idispharma.com).

If falciparum malaria is acquired at the Cambodia–Thailand border region, artesunate resistance should be considered; except for this region, where mefloquine resistance also is a problem, artesunate is considered to be an efficacious drug with limited reports of resistance. In conclusion, the current case series suggests that IV artesunate is an efficacious and safe treatment option in travelers returning from West Africa with severe falciparum malaria.
